# Retinal vascular tortuosity assessment: inter-intra expert analysis and correlation with computational measurements

**DOI:** 10.1186/s12874-018-0598-3

**Published:** 2018-11-20

**Authors:** Lucía Ramos, Jorge Novo, José Rouco, Stephanie Romeo, María D. Álvarez, Marcos Ortega

**Affiliations:** 1 University of A Coruña, Department of Computer Science, Campus de Elviña, A Coruña, 15071 Spain; 2 CITIC-Research Center of Information and Communication Technologies, University of A Coruña, A Coruña, Spain; 3 Servizo de Oftalmoloxía, Complexo Hospitalario Universitario de Ferrol, Ferrol, A coruña, Spain

**Keywords:** Retinal circulation, Vascular tortuosity, Fundus images, Computer-aided diagnosis, Image analysis

## Abstract

**Background:**

The retinal vascular tortuosity can be a potential indicator of relevant vascular and non-vascular diseases. However, the lack of a precise and standard guide for the tortuosity evaluation hinders its use for diagnostic and treatment purposes. This work aims to advance in the standardization of the retinal vascular tortuosity as a clinical biomarker with diagnostic potential, allowing, thereby, the validation of objective computational measurements on the basis of the entire spectrum of the expert knowledge.

**Methods:**

This paper describes a multi-expert validation process of the computational vascular tortuosity measurements of reference. A group of five experts, covering the different clinical profiles of an ophthalmological service, and a four-grade scale from non-tortuous to severe tortuosity as well as non-tortuous / tortuous and asymptomatic / symptomatic binary classifications are considered for the analysis of the the multi-expert validation procedure. The specialists rating process comprises two rounds involving all the experts and a joint round to establish consensual rates. The expert agreement is analyzed throughout the rating procedure and, then, the consensual rates are set as the reference to validate the prognostic performance of four computational tortuosity metrics of reference.

**Results:**

The Kappa indexes for the intra-rater agreement analysis were obtained between 0.35 and 0.83 whereas for the inter-rater agreement in the asymptomatic / symptomatic classification were between 0.22 and 0.76. The Area Under the Curve (AUC) for each expert against the consensual rates were placed between 0.61 and 0.83 whereas the prognostic performance of the best objective tortuosity metric was 0.80.

**Conclusions:**

There is a high inter and intra-rater variability, especially for the case of the four grade scale. The prognostic performance of the tortuosity measurements is close to the experts’ performance, especially for Grisan measurement. However, there is a gap between the automatic effectiveness and the expert perception given the lack of clinical criteria in the computational measurements.

## Background

In recent years, medical imaging has become crucial in the clinical decision-making process, playing an important role to improve the public health due to its ability to extract information for diagnosis and treatment purposes. The use of large databases for medical imaging also implies the challenge of handling such amount of information in a reliable and useful way for the clinical expert. In addition to this, many medical imaging-based procedures present low repeatability, mainly due to the subjective appreciation of the analyzed data, the variability of the image conditions, or even the expert training for a specific task. Besides the subjectivity, the manual characterization of a large image dataset is a tedious and time-consuming task that inevitably leads to a decreasing performance over time for the same expert. In that sense, the use of computer-based systems that provide the image storage and analysis by a common repeatable procedure allows ensuring an objective and reliable environment for the specialists, improving, thereby, the productivity and efficiency of the clinical performance.

In opthalmology, retinal image analysis is an useful tool for the noninvasive diagnosis of many relevant diseases, such as hypertension, diabetes or atherosclerosis. Common symptoms of those pathologies include neovascularization, occurrence of pathological structures, or increased tortuosity that can be observed analyzing the vascular tree of the eye fundus. Given the importance of the eye fundus study, Sirius (System for the Integration of Retinal Images Understanding Services) was proposed in [1] as a computer aided diagnosis tool for the analysis of retinal images. It provides a framework for ophthalmologists or other experts to collaboratively work using retinal image-based applications in a distributed, fast and reliable environment. Sirius integrates several image processing algorithms structured as independent modules. One of the modules is in charge of the automatic arterio-venous ratio (AVR) calculation [2], a relevant biomarker to determine the vascular risk that is associated to diseases that affect the circulatory system such as hypertension. Another module localizes microaneurysms [3], which are small red points that appear in early stages of diabetic retinopathy. A third module is focused on measuring the vascular tortuosity of the blood vessels [4, 5], that is, how and how many times a vessel bends, complementary to the AVR parameter. It is a indicator for a number of vascular and nonvascular diseases such as diabetic retinopathy, cerebrovascular disease, stroke, and ischemic heart disease [6–9]. This module integrates four different metrics of tortuosity of reference [10–13].

The validation of Sirius modules against the manual evaluations performed by clinical experts is crucial to ensure a repeatable and reliable analysis of the biomedical parameters that are extracted from the retinal images. The AVR prognostic value, as computed in Sirius, has been clinically validated by Pose et al. [14]. The posterior validation of this module has been carried out in different real environments involving several health care systems [1]. Moreover, additional evaluations of Sirius vessel width measurement have been conducted in DRIVE and REVIEW databases [15, 16]. Regarding the tortuosity module, a preliminary validation over a set of retinal images previously classified as tortuous / non-tortuous has been presented by Sánchez et al. [4, 5].

Although retinal vascular tortuosity is underlying both vascular and systemic diseases, its manual characterization is affected by several limitations that still restrict its use to research purposes. Systematic reviews of retinal vessel tortuosity measures and clinical findings related to them conducted by Kalitzeos et al. [17] and Abdalla et al. [18] compile the main limitations for using the retinal vascular tortuosity as a clinical marker for diagnostic, treatment and monitoring purposes. One of the main limitations is the lack of a precise and standard guide for the tortuosity assessment regarding the image acquisition, measurement location and consequent calculation. In the clinical practice, the manual characterization of the retinal vascular tortuosity is mostly based on clinical experience by identifying relative characteristics such as the dissimilarity to normal healthy vessels in terms of length, width or number of twists, among others, also evaluating changes in and around each vessel. Therefore, the grading is performed on a subjective scale resulting in a tedious and time-consuming task with a remarkable inter and intra expert variability. Another aspect stated in these reviews is that different diseases produce different tortuosity effects [9, 19, 20], so that the vascular tortuosity should be analyzed from each specific pathological point of view. Despite this, the absence of unified public datasets, the limited size of the existing ones or the differences in the segmentation techniques for extracting blood vessels and the medical state of the patients at the moment of screening, hinder the validation processes of available computational measurements. Additionally, most computational metrics are depending on one or two factors such as the curvature or the number of twists. However, the experts, based on their experience, consider additional parameters such as dilation, elongation, vessel calibers or branching angles [21, 22], among others, that are non incorporated in the current computational metrics of reference. The limitations extracted from these reviews indicate the necessity for standardizing the image acquisition, parameter calculation and analysis of the retinal vascular tortuosity in order to become more useful and reliable to support the clinical decision-making processes.

In the work herein described, a complete and exhaustive multi-expert validation procedure for the Sirius tortuosity module is proposed. This study aims, first, to lay the foundations for advancing the standardization of the retinal vascular tortuosity as a clinical biomarker with diagnostic potential. Once a consistent clinical criteria is established, the validation of the prognostic performance of objective computational measurements of reference is performed.

In order to cover the entire spectrum of the expert knowledge, the validation experiments included a group of five different experts with gradual levels of expertise that usually work in a ophthalmological service of the health care systems, from the head of the service to resident physicians. The manual rating was performed on the basis of a four-grade qualitative scale from non-tortuous to severe tortuosity, being complemented with non-tortuous / tortuous and asymptomatic / symptomatic binary classifications. A rating procedure divided in several rounds was designed in order to set a consensual ground-truth and the extraction of uniform criteria. To this end, first, the five experts rated separately the whole dataset in a blind process. In order to gain consensus, the discrepancies were analyzed followed by a second rating round that was carried out by each expert. Finally, a joint session involving all the experts was held to set total consensual rates. Therefore, the expert agreement was analyzed throughout the rating procedure and, then, the consensual rates were set as reference to compare the individual manual and automatic measurements. This way, the prognostic performances of the tortuosity metrics presented in [4] were evaluated in relation to the experts performance.

This paper is organized as follows: “Materials and methods” section describes the designed dataset, the details of the automatic tortuosity metrics and the procedure for the multi-expert validation. Next, Section Results exposes all the conducted experiments and Section Discussion discusses the obtained results and the constraints and potential of the tortuosity characterization. Finally, “Conclusions” section presents the conclusions and possible future work.

## Materials and methods

### Dataset and rating procedure

Given the association of the retinal vascular tortuosity with diabetes and, specifically, diabetic retinopathy [23–25], fundus images from diabetic patients were found representative for this study. Although vascular tortuosity is underlying more pathologies, different diseases present different tortuosity effects [18]. Given the limited cohort of patients, in order to incorporate homogeneous data, the dataset is limited to diabetic patients ranging from none, mild, moderate, severe or proliferative diabetic retinopathy in a balanced distribution. Therefore, the designed dataset consists of 60 fundus images varying from non visible anomalies in the vessels to severe tortuosity.

In order to analyze the intra and inter-rater variability, a group of five experts belonging to different levels of an ophthalmological service, from the head of the service to resident physicians, manually rated these images. Therefore, the manual characterization of the retinal vascular tortuosity covers the entire spectrum of the expert knowledge. In the clinical practice, ophthalmologists commonly evaluate the degree of a retinal blood vessel tortuosity by considering changes in and around that vessel. These signs are mostly based on clinician experience and knowledge since there is no standard guide for the tortuosity evaluation. Given the lack of standard, the experts, on the basis of their experience, decided to use initially a qualitative four-grade scale which comprises none, mild, moderate, and severe tortuosity degree. Throughout the rating procedure, the experts self-instructed themselves and jointly decided the use of two binary classifications obtained by grouping the grades according to their association with meaningful clinical conditions. In one hand, a classification to discriminate between no sign of tortuosity and any tortuosity level from mild to severe is considered. It is equivalent to the tortuous / non-tortuous classification used in previous validation experiments [4, 5]. On the other hand, with the knowledge that mild tortuosity is asymptomatic whereas moderate and severe tortuosity can lead to significant risks [22], the classification asymptomatic / symptomatic is defined by grouping none and mild grades in one class and moderate and severe grades in the other class. Before and during the rating procedure, the experts were only instructed with the explicit indication of sticking to the evaluation of vascular tortuosity, abstracting from other clinical findings in the fundus images that could bias the manual rating. For the same reason, the information related to the patient medical state was not available for the experts. The rating procedure was carried out through the progressive following steps:

- A first rating round (*R*_1_) using the four-grade scale involving all the experts separately, in a totally blind process.
- A meeting with the experts to discuss the discrepancies and clarify the criteria for the next round. In order to have a control expert that preserves the initial criteria, the expert *E*_5_ did not attend to this meeting.
- A second rating round (*R*_2_) for each expert using asymptomatic / symptomatic classification, since after the meeting it is selected as more relevant for the clinical practice.
- A joint round (*R*_*c*_) to set unified consensual rates for the asymptomatic / symptomatic classification.

Table 1 summarizes the manual rates that were provided by the set of experts *E*={*E*1,*E*2,..,*E*5} for the whole dataset using the four-grade scale in the initial blind rating round *R*_1_, grouped by grading.

**Table 1:** Retinal vessel tortuosity rated by 5 experts in the first round using a four-grade scale

Grading	*E*1_*R*1_	*E*2_*R*1_	*E*3_*R*1_	*E*4_*R*1_	*E*5_*R*1_
0:none	34	10	21	20	31
1:mild	19	29	23	25	18
2:moderate	7	20	16	12	11
3:severe	0	1	0	3	0

In addition to this grading, these rates were grouped according to the tortuous / non-tortuous and the asymptomatic / symptomatic binary classifications. After the meeting with the experts for analyzing these rates, the binary classification asymptomatic / symptomatic was set as the most relevant for the clinical practice due to the need for medical treatment in symptomatic cases. Table 2 summarizes the rates for the asymptomatic / symptomatic classification obtained by grouping the labels of *R*_1_ and from the manual rates of *R*_2_.

**Table 2:** Retinal vessel tortuosity rated by 5 different experts in *R*_1_ and *R*_2_ using a classification asymptomatic / symptomatic

		E1	E2	E3	E4	E5
R1	0:none-mild	53	39	44	45	49
	1:moderate-severe	7	21	16	15	11
R2	0:none-mild	37	39	34	27	50
	1:moderate-severe	23	21	26	33	10

In order to extract a global expert assessment and discard some isolated dissenting cases, the most voted rates for each image are analyzed in the rounds *R*_1_, *R*_2_, as well as the set formed by the union of *R*_1_ and *R*_2_. Therefore, for each image, *V*_*R*1_ is set to the most voted rate in the round *R*_1_, *V*_*R*2_ is the most voted rate in the round *R*_2_ and *V*_*R*1*R*2_ is the most voted rate in the set including all the rates in rounds *R*_1_ and *R*_2_. Finally, in order to clarify the debatable cases and set unified consensual rates, a joint *R*_*c*_ was carried out involving the five experts. Table 3 summarizes the rates from $V_{R_{1}}$, $V_{R_{2}}$, $V_{R_{1}R_{2}}$ and *R*_*c*_.

**Table 3:** Retinal vessel tortuosity rates from $V_{R_{1}}$, $V_{R_{2}}$, $V_{R_{1}R_{2}}$ and *R*_*C*_ using the asymptomatic / symptomatic classification

	*V*_*R*1_	*V*_*R*2_	*V*_*R*1 *R*2_	*R* *c*
0:none-mild	52	37	45	44
1:moderate-severe	8	23	15	16

#### Automatic measurement of the retinal vessel tortuosity

**Fig. 1:**
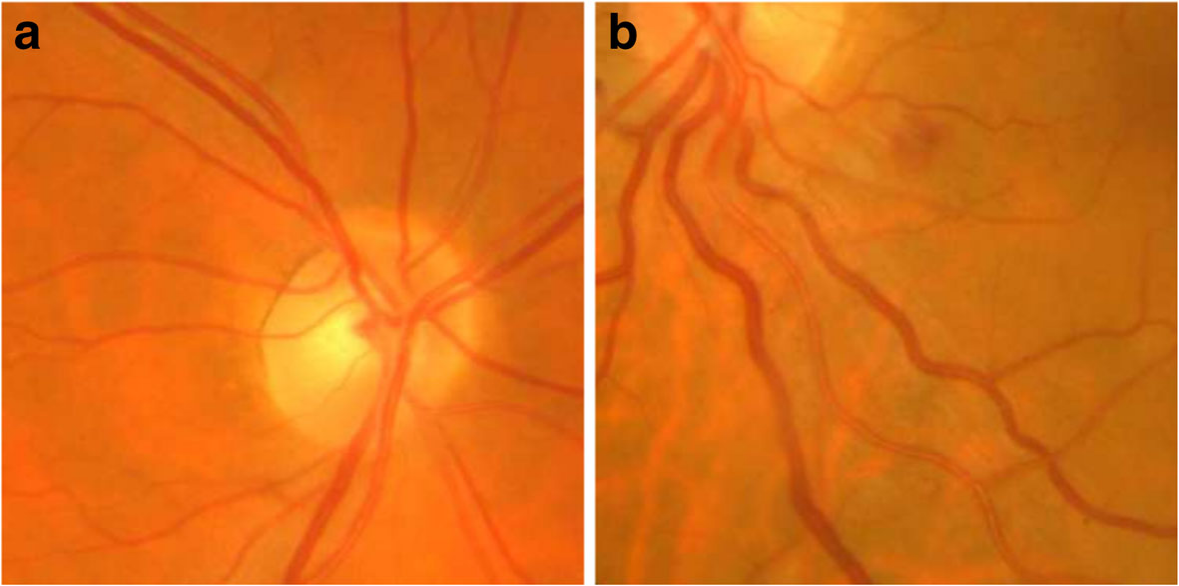
Retinal images **a** Non-tortuous and **b** tortuous blood vessels

Retinal blood vessels are normally straight or slightly and gradually curved. However, vascular diseases can cause tortuosity in its structure, defined as a non-smooth appearance of the vessel course. Tortuosity may affect to a small region or involve the entire retinal vascular tree. Figure 1 shows representative examples of retinal images with non-tortuous and tortuous blood vessels.

**Fig. 2:**
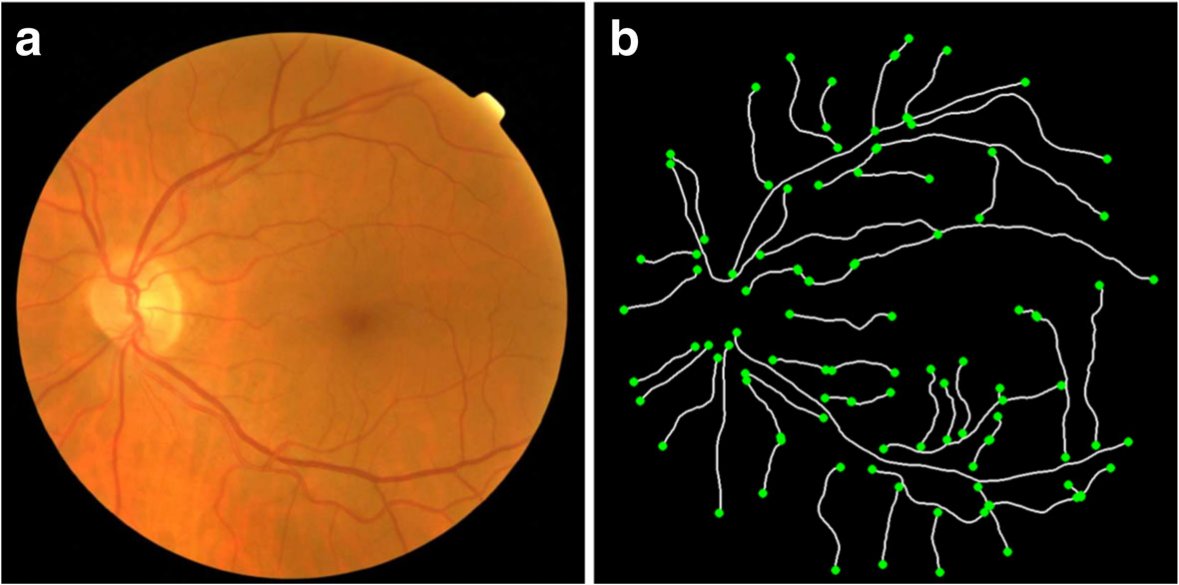
Arterio-venous tree segmentation. **a** Original retinal image. **b** Ends of each vessel of the segmented vascular tree

Based on the computational metrics of reference, all the vessels composing the retinal vascular tree are involved in the tortuosity computation. Therefore, given a color retinal image (see Fig. 2a), the first step to automate the tortuosity measurement consists of the extraction of the arterio-venous tree, and then, its division into the composing vessels. Then, a tortuosity value is computed for each of these vessels, and finally, the tortuosity values corresponding to all the vessels are integrated in a total tortuosity value that is associated with the whole retina.

To this end, the retinal vessels are extracted, first, from an algorithm based on the crease extraction [26, 27]. This algorithm consists in detecting the blood vessels from the ridges or valleys in the retinal image, this is, regions that form an extreme and tubular level on the neighborhood. Therefore, the Multi Local Set of Extrinsic Curvature enhanced by the Structure Tensor (MLSEC-ST) operator is applied to detect the vessels from the ridge lines. Then, a thinning process is performed to extract the centerline of a maximum of 1*px* width for each vessel [28]. After this, an edge tracking algorithm is applied to decompose the vessel tree into its constituent vessels. Finally, the vessel point coordinates are locally smoothed in order to minimize the discrete effect of the pixel representation. The resulting vessel segments are used for the tortuosity computation (see Fig. 2b).

Using the vessel segments as reference, four different metrics for measuring the tortuosity of retinal vessels were considered:

#### First approach - Hart et al. [10]

The first approach, proposed by Hart et al. [10], is the simplest and most widely used measure. It computes the tortuosity of a vessel by examining how long the curve is relative to its chord length, as follows:

1 $$ \tau_{Hart}=\frac{L_{c}}{L_{x}} - 1   $$

where *L*_*c*_ is the arc length or curve length obtained by counting all the points from the start of the vessel to its end, and *L*_*x*_ is the length of the underlying chord, that is, the euclidean distance between the two end points of the vessel.

#### Second approach - Grisan et al. [11]

Another tortuosity metric has been proposed by Grisan et al. [11]. This approach subdivides each vessel in *n* segments of constant-sign curvature and then combines the evaluation of such segments and their number as follows:

2 $$ \tau_{Grisan}=\frac{n-1}{L_{c}}\sum\limits_{i=1}^{n}\left[\frac{L_{csi}}{L_{xsi}}-1\right]   $$

where *L*_*c*_ corresponds to the arc length of the vessel whereas *L*_*csi*_ and *L*_*xsi*_ represent the arc length and the chord length of each subsegment. This metric integrates the information about how many times a vessel changes convexity so that a higher number of subsegments implies higher tortuosity.

#### Third approach - Trucco et al. [12]

The third approach implements the proposal of Trucco et al. [12], that consists of a measure only depending on the vessel skeleton curvature. This metric is a generalized version of the curvature based metrics presented in Hart et al. [10]. It is defined by:

3 $$ \tau_{Trucco}=\left(\sum\limits_{j}\left|k_{s}(j)\right|^{p}\right)^{\frac{1}{p}}   $$

where *p* is a strictly positive integer and *k*_*s*_(*j*) is the curvature at the *jth* point of the vessel *s*, defined as follows:

4 $$ k_{s}(j)=\frac{x^{\prime}(j)y^{\prime\prime}(j)-x^{\prime\prime}(j)y^{\prime}(j)}{\left[y^{\prime}(j)^{2}+x^{\prime}(j)^{2}\right]^{3/2}},   $$

The curvature measures the variation of the slope of the line tangent to the curve at each point along the segment. A significant difference in slope between the point and its surrounding neighbors implies a high curvature.

#### Fourth approach - Onkaew et al. [13]

Finally, the system implements the metric proposed by Onkaew et al. [13], that uses the number of points where the curvature changes its sign, but this curvature is calculated from a improved chain-code algorithm. This method labels each point using its relative position in relation to the previous point, traveling all the intermediate points along the vessel. The tortuosity metric is defined as follows:

5 $$ \tau_{Onkaew}=\frac{n-1}{n}\frac{1}{L_{c}}\sum\limits_{i=1}^{n}K\left(p_{i},k\right)   $$

where *L*_*c*_ corresponds to the arc length of the vessel, *n* is the number of subsegments composing the vessel and *K*(*p*_*i*_,*k*) is the curvature at each point computed by using the chain-code algorithm.

#### Integration of the vessel tortuosity values

The proposed metrics allow the tortuosity calculation from each particular vessel. Once the tortuosity values are computed for each vessel composing the vascular tree, these values are integrated in a total tortuosity measurement using their weighted average. This way, the computed total tortuosity is associated as a single score to the whole retina. Therefore, using the compositionality property of the proposed measures in (1), (2), (3) and (5), each vessel contributes inversely proportional to its arc length [10]. This weighted additivity is defined as:

6 $$ \tau(c1,c2)=\frac{\left[L_{c1}\tau_{c1}+L_{c2}\tau_{c2}\right]}{L_{c1}+L_{c2}}   $$

where *L*_*ci*_ is the arc length of the vessel *ci* and *τ*_*ci*_, the tortuosity value for that vessel. Therefore, the resulting tortuosity is within the range of the constituent vessels [11]. These metrics have a dimension of 1/*lenght* and thus may be interpreted as a tortuosity density, allowing the comparison between retinal images at different scales.

### Expert agreement analysis

An overall comparison including all the manual rates for the whole dataset was performed in order to evaluate the expert agreement and set a reference for validating the performance of the computational approaches. To this end, the percentages of retinal images with full consensus and with four or at least three expert coincidences were extracted for the four-grade scale and for the tortuous / non-tortuous and asymptomatic / symptomatic binary classifications in rounds *R*_1_ and *R*_2_.

Then, an extended analysis was performed for the asymptomatic / symptomatic classification since the discussion between experts throughout the rating process concluded that this classification is more relevant for the clinical practice. For this purpose, Cohen-Kappa indexes [29] were computed between each pair of experts in rounds *R*_1_ and *R*_2_ in order to evaluate the intra and inter-rater agreement for this classification. Moreover, the agreement in relation to *V*_*R*1_, *V*_*R*2_, *V*_*R*1*R*2_, and the consensus session *R*_*c*_ was also analyzed.

### Multi-expert validation analysis

In order to evaluate the prognostic performance of a tortuosity metric, a ROC (Receiver Operating Characteristic) analysis was carried out by using the asymptomatic / symptomatic classification as target prediction. To this end, ROC curves were built from the reciprocal relation between sensitivity and specificity calculated for all the possible threshold values in the automatic metrics [30]. Thus, tortuosity metrics can be evaluated against each of the expert predictions. This same ROC analysis can be used to evaluate the performance of each expert against the others, but in this case, only one point of the ROC space is obtained.

With the aim of setting a consistent ground-truth that includes all the unified criteria that were extracted throughout the rating process, the consensual rates in *R*_*c*_ are set as the reference to evaluate the performance of the different tortuosity metrics. Therefore, $\overline {ROC}(Ei_{Rj}, R_{c})$ represent the point in the space ROC for expert *i* in round *j* respect to *R*_*c*_. Similarly, $\overline {ROC}(\tau _{m}, R_{c})$ corresponds to the ROC curve for tortuosity metric *τ*_*m*_ in relation to *R*_*c*_. Comparing the AUC values of the equivalent curves allow to evaluate if the prognostic performance is similar to the experts performance.

## Results

### Expert agreement results

**Fig. 3:**
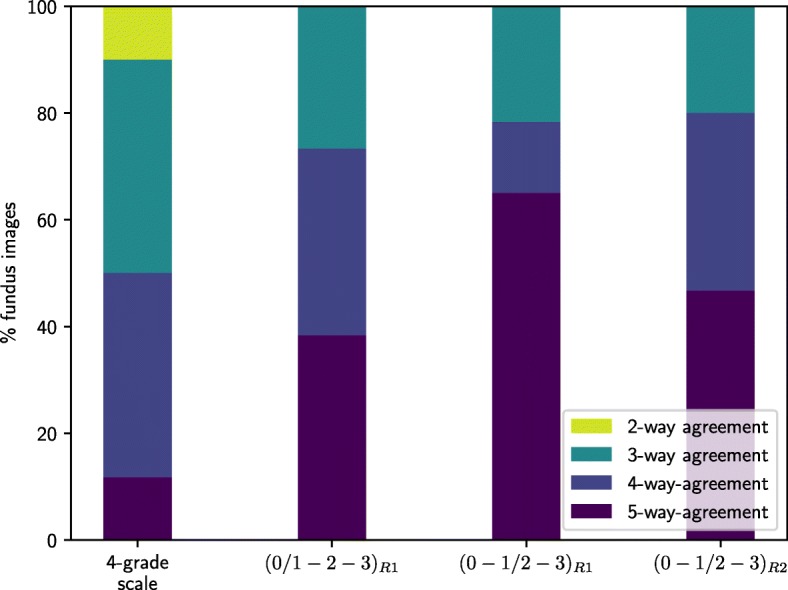
Overall comparison among all the manual rates in the whole dataset. Expert agreement for four-grade, (0 / 1-2-3) and (0-1 / 2-3) scales in rating rounds *R*_1_ and *R*_2_

An overall comparison was performed among all the manual rates in the whole dataset for the four-grade scale and also for the non-tortuous / tortuous and asymptomatic / symptomatic classifications in rounds *R*_1_ and *R*_2_. Figure 3 shows the percentages of retinal images with full consensus among all the experts, the percentages where there are four coincidences, and the percentages where, at least, three experts agree in their labels.

Complementarly, Table 4 shows the Cohen-Kappa indexes between each pair of experts in rounds *R*_1_ and *R*_2_ and between each expert and *V*_*R*1_, *V*_*R*2_, *V*_*R*1*R*2_, and the consensus session *R*_*c*_ for asymptomatic / symptomatic classification. An standard guideline for interpreting these indexes [31] assumes slight agreement from 0.0 to 0.20, fair agreement from 0.21 to 0.40, moderate agreement from 0.41 to 0.60, substantial agreement from 0.61 to 0.80 and almost perfect or perfect agreement for values greater than 0.81. The Cohen-Kappa indexes that are highlighted in bold correspond to the intra-rater agreement between rounds *R*_1_ and *R*_2_. The rates in *R*_*c*_ are selected as the reference to validate the prognostic performance of the tortuosity automatic measurements since they represent the consensual criteria concluded throughout the rating process.

**Table 4:** Inter-intra expert agreement analysis

Cohen-Kappa	*E*2_*R*1_	*E*3_*R*1_	*E*4_*R*1_	*E*5_*R*1_	*E*1_*R*2_	*E*2_*R*2_	*E*3_*R*2_	*E*4_*R*2_	*E*5_*R*2_		*V*_*R*1_	*V*_*R*2_	*V*_*R*1 *R*2_	*R*_*c*_
**E**1_*R*1_	0.39	0.53	0.57	0.61	**0** **.** **3** **5**	0.39	0.22	0.20	0.66		0.92	0.35	0.57	0.53
**E**2_*R*1_		0.50	0.53	0.42	0.43	**0** **.** **5** **6**	0.34	0.35	0.38		0.44	0.43	0.53	0.42
**E**3_*R*1_			0.43	0.48	0.44	0.50	**0** **.** **5** **0**	0.33	0.52		0.59	0.51	0.70	0.74
**E**4_*R*1_				0.51	0.55	0.61	0.39	**0** **.** **4** **3**	0.45		0.63	0.55	0.64	0.70
**E**5_*R*1_					0.37	0.51	0.31	0.25	**0** **.** **8** **3**		0.69	0.45	0.71	0.57
**E**1_*R*2_						0.50	0.76	0.48	0.23		0.40	0.86	0.62	0.66
**E**2_*R*2_							0.48	0.48	0.46		0.44	0.64	0.69	0.73
**E**3_*R*2_								0.57	0.34		0.26	0.83	0.54	0.57
**E**4_*R*2_									0.28		0.22	0.61	0.43	0.46
**E**5_*R*2_											0.74	0.49	0.65	0.52
*V*_*R*1_												0.40	0.63	0.59
*V*_*R*2_													0.70	0.74
*V*_*R*1 *R*2_														0.87

Cohen-Kappa indexes for intra and inter-rater agreement as well as between each expert and *V*_*R*1_, *V*_*R*2_, *V*_*R*1*R*2_ and *R*_*c*_ in rounds *R*_1_ and *R*_2_ for asymptomatic / symptomatic classification. The indexes that are highlighted in bold correspond to the intra-rater agreement between rounds *R*_1_ and *R*_2_

### Multi-expert validation results

**Fig. 4:**
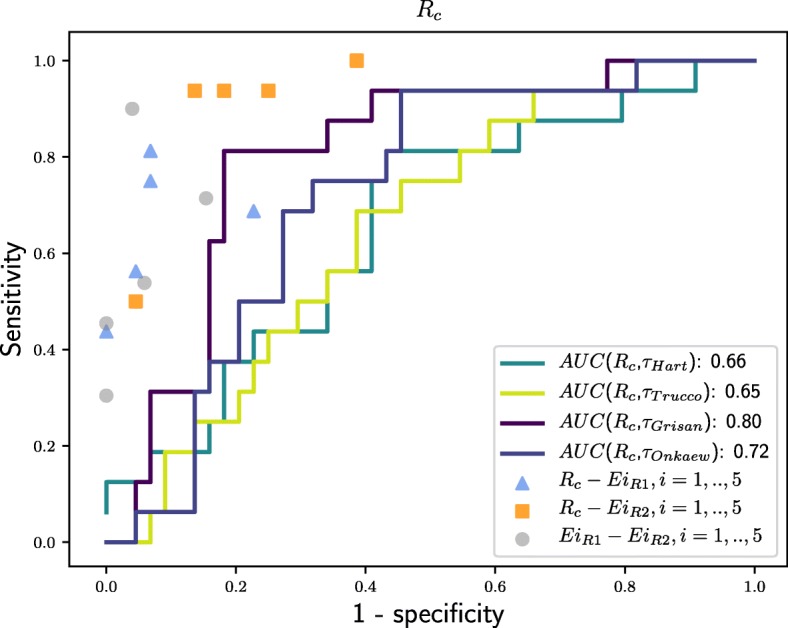
Prognostic performance of retinal vascular tortuosity measurements. ROC curves to evaluate prognostic performance of automatic tortuosity measurements in relation to *R*_*c*_

The consensual rates from *R*_*c*_ are used for validating the prognostic performance of tortuosity measurements. To this end, the points in the ROC space $\overline {ROC}(Ei_{Rj}, R_{c})$ for *i*=1..5,*j*=1,2 are computed as shown in Fig. 4 represented by triangular and square marks. In the same way, the ROC curves $\overline {ROC}(\tau _{m}, R_{c})$ for each tortuosity metric in relation to *R*_*c*_ are built. Moreover, the intra-expert reliability, represented as circular marks, is also considered.

## Discussion

The results extracted from the overall comparison among the manual rates show that there is a high inter-rater variability, especially for the four-grade scale. Regarding the binary classifications, the experts agree with higher rates in the discrimination between asymptomatic / symptomatic than between non-tortuous / tortuous retinal images. In the rating round *R*_2_, the percentage of images with full consensus decreased mostly due to the rates of *E*_5_, the control expert that did not attend to the meeting to discuss the discrepancies and keep its initial criteria, indicating, thereby, the utility and suitability of the meeting. Hence, there is a slight increment in the percentage of images where at least four expert converge since the discussion allowed to unify criteria and gain consensus.

Regarding the Cohen-Kappa indexes, they show low or fair agreement for the four experts who attended the session to clarify the discrepancies found in *R*_1_, since after the meeting, they change their criteria for the second round *R*_2_. However, *E*_5_, the control expert who was not involved in that session, made a similar rating in both rounds, given its criteria was not influenced and modified, presenting, thereby, a high intra-rater agreement. According to the data showed in Table 2, in the round *R*_1_, the experts were more conservative for asymptomatic cases whereas in the round *R*_2_ the sensitivity for symptomatic cases increased. The change in the criteria is mainly due to the fact that initial rates corresponds to a global assessment of the whole retina, mostly focused on the main vessels, nevertheless, the expert meeting for analyzing the discrepancies led to a more local analysis taking into account each specific vessel during the round *R*_2_. The criteria refinement is also reflected in the low index between *V*_*R*1_ and *V*_*R*2_. However, the rates obtained by combining *R*_1_ and *R*_2_ are quite close to the consensual rates in *R*_*c*_ since *V*_*R*1*R*2_ represents the majority inclination comprising the conservative criteria based in the global perception followed in *R*_1_ as well as the analysis of specific vessels considered in *R*_2_.

With respect to the objective tortuosity measurements, Fig. 4 shows that the prognostic performance is below, at different distances, of the experts performance. As detailed before, the analyzed computational metrics incorporate parameters as amplitude, number of twists or curvature of the retinal blood vessels, depending on each case. The results show that the best performance is provided by the metrics which integrate the information about how many times a vessel changes its convexity. In particular, the metric that reached the best score was the Grisan proposal, followed by the Onkaew proposal, given they combine the number of segments with constant convexity within a vessel with the evaluation of such segments. However, Hart and Trucco proposals analyze each vessel globally, regardless of whether it has a constant sign or presents twists.

### Tortuosity characterization. Constraints and potential

The assessment of the retinal vascular tortuosity is affected by several factors that prevent its use for diagnostic and treatment purposes. Thus, the lack of precise and standard guides for tortuosity characterization leads to a remarkable disagreement among the experts. In this sense, the multi-expert validation process throughout a rating procedure in several stages is raised in order to lay the foundations for advancing the standardization of the retinal vascular tortuosity as a potential indicator for diagnostic purposes.

Besides the subjective appreciation of tortuosity signs, the manual characterization is also depending on the experience of the rater. In order to cover the entire spectrum of the expert knowledge, a group of five clinicians belonging to different levels of an ophthalmological service was considered for the rating procedure. In particular, they cover the head of the service, experienced clinicians with different levels of expertise and also the participation of resident physicians. This way, the manual characterization of the retinal vascular tortuosity incorporates assessments at different levels of expertise and medical profiles. In order to avoid biased rates, the information related to the patient medical state was not known by the experts at the time of the manual rating. The rating procedure was performed individually in a totally blind process in which the experts were only instructed with the explicit indication of sticking to the evaluation of the vascular tortuosity, abstracting from other clinical findings that could bias the rating.

Despite this, there are also limitations related to the availability of normative data due to the absence of unified public datasets. Moreover, even the available datasets, public or private, present limitations in terms of type and size. This, along with the lack of a standard regarding the computational algorithms used for extracting the blood vessels or the location of the tortuosity measurements, hinder the validation processes of the available computational methods. Furthermore, different diseases produce different tortuosity effects, so that the vascular tortuosity should be analyzed from each specific pathological point of view. In this work, given the association of the retinal vascular tortuosity with diabetes and, more specifically, diabetic retinopathy, diabetic patients were found representative for this study. Although vascular tortuosity is underlying more pathologies, the dataset was limited to diabetic patients in order to analyze a representative cohort of homogeneous data. With respect to the type and size of the retinal images, the implemented methods allow a high degree of normalization in the computed tortuosity values, independently of the acquisition procedure. Regarding the location or zone of the vessels involved in the tortuosity computation, this analysis is based on the metrics of reference in the literature [10–13], included in the Sirius framework [1]. According to these metrics, the vascular tree is extracted by means of a consolidated computational methodology [26] for that purpose, being all the vessels composing the vascular tree involved in the global tortuosity computation.

Regarding the prognostic performance of the computational metrics, despite the acceptable results of some of the metrics, all of them remain at a distance of the experts performance. The metrics of reference generally use mathematical properties depending on one or two factors such as curvature or number of twists. However, the experts, based on their experience, analyze a larger set of properties being, therefore, differentiated of the computational metrics.

## Conclusions

The retinal vascular tortuosity constitutes a potential indicator of relevant vascular and non-vascular diseases, so that a reliable quantitative measurement would be a potential biomarker for early detection and disease prevention. However, there is no a precise and standard definition of the vascular tortuosity, and consequently, its manual characterization is a subjective task with a high variability. This work is raised with the aim of establishing the basis for advancing in the standardization of the retinal vascular tortuosity as a clinical marker with diagnostic potential allowing, thereby, the robust validation of computational measurements to ensure an objective and reliable environment for the retinal experts. For this purpose, a multi-expert validation procedure is presented in order to assess the prognostic performance of the computational calculation of the vascular tortuosity following the main referenced strategies, included in the Sirius framework. The presented validation included the participation of a group of five different experts and considered a four-grade scale from non-tortuous to severe tortuosity as well as non-tortuous / tortuous and asymptomatic / symptomatic binary classifications. The rating procedure comprised 2 rating rounds in which each expert manually rated the whole dataset and a posterior final joint consensus session where the debatable cases were discussed to reach a global agreement.

For the multi-expert validation procedure, firstly, the expert agreement was analyzed along the different rating rounds. The intra and inter-rater reliability were computed and the discrepancies were discussed involving the experts in order to clarify the criteria and extract additional information from their clinical perception. This rating process allowed to gain consensus among the experts and get consensual rates comprising all the unified criteria extracted throughout the rating process. Therefore, the consensual rates were set as a reference for validating the computational tortuosity metrics that were included in this analysis. Once a consolidated clinical criteria was established, the prognostic performance of the computational measurements was compared to the experts performance, allowing a robust validation of the strengths and limitations of the different tortuosity metrics of reference.

The multi-expert validation provided acceptable results, especially regarding the Grisan proposal. However, all of the considered computational measurements remain at a distance of the experts performance. The analyzed metrics use mathematical properties to define the degree of tortuosity according to one or two factors such as as the amplitude, the curvature or the number of twists, depending on each case. Despite that, the experts, based on their experience, analyze additional parameters such as the neovascularization, the vessel caliber or the distinction between arteries and veins that are not incorporated, at the moment, in the existing computational metrics, thereby causing differences between the automated effectiveness and the expert perception. The results extracted from this work demonstrate that the metrics of reference do not provide a full representation of the expert perception so that additional parameters should be incorporated in the computational metrics in order to have a more accurate and reliable tortuosity assessment. Thus, future work in this research line includes the integration of additional properties in new computational proposals that could approach the performance of the computational metrics to the knowledge of the expert clinicians.

## Abbreviations

AUC: Area under curve
AVR: Arterio-venous ratio
ROC: Receiver operating characteristic
Sirius: System for the integration of retinal images understanding services
